# Nano-laponite/polymer composite as filtration reducer on water-based drilling fluid and mechanism study

**DOI:** 10.1098/rsos.220385

**Published:** 2022-10-12

**Authors:** Xiaodong Dong, Jinsheng Sun, Xianbin Huang, Kaihe Lv, Zhishi Zhou, Chongyang Gao

**Affiliations:** ^1^ School of Petroleum Engineering in China University of Petroleum (East China), Qingdao 266580, People's Republic of China; ^2^ Key Laboratory of Unconventional Oil & Gas Development, Ministry of Education, Qingdao 266580, People's Republic of China; ^3^ Petro China Tarim Oilfield Company, Korla, Xinjiang 841000, People's Republic of China

**Keywords:** nano-laponite, fluid loss reducer, water-based drilling fluid, high-temperature resistance, saturated salt resistance

## Abstract

In drilling deep complex formations, most drilling fluid additives have insufficient temperature and salt tolerance, resulting in the decline of drilling fluid performance. This study used 2-acrylamide-2-methylpropane sulfonic acid, acrylamide, dimethyl diallyl ammonium chloride and modified nano-laponite to synthesize a nanocomposite filtrate reducer (ANDP) with excellent temperature and salt resistance, which can maintain the performance of drilling fluid. The structure of ANDP was analysed by a transmission electron microscope and an infrared spectrometer. The thermal stability of ANDP was studied by thermogravimetric analysis. The performance of ANDP was evaluated in a water-based drilling fluid. The mechanism was analysed per clay particle size distribution, Zeta potential, filter cake permeability and scanning electron microscopy imaging. The results show that ANDP has good thermal stability and the expected molecular structure. The filtration of freshwater drilling fluid after ageing at 200°C is 10.4 ml and that of saturated brine drilling fluid is 6.4 ml after ageing at 150°C. Mechanism analysis suggests that the ANDP increases the thickness of clay particle hydration layer and maintains the colloidal stability of the drilling fluid. ANDP inhibits the agglomeration of clay particles and significantly reduces the filtration by forming dense mud cake.

## 1. Introduction

The growing demand for oil resources by China's energy industry is hampered by the gradual reduction of its shallow conventional oil and gas reserves. This has forced the exploration for domestic oil and gas resources to develop toward deeper strata. Unconventional oil and gas resources such as tight oil, shale oil and shale gas have become the focus of development [1–4]. Fortunately, China's deep oil resources in deep strata are extensive and widely distributed. Recent discoveries of large oil and gas reservoirs are distributed mainly in deep and ultra-deep strata—the depth of which can exceed 6000 m. With the need to drill deeper, the danger of striking complex formations such as high-temperature, high-pressure and salt gypsum layers also increases, which results in higher requirements and greater challenges for deep and ultra-deep drilling technology, as well as for deep and ultra-deep drilling fluid technology [5–8].

Drilling fluid is the fluid that circulates during drilling and has various functions. It has always been called the ‘blood of drilling’. Drilling fluid plays an essential role in putting in suspension and carrying cuttings, stabilizing wellbore, balancing formation pressure, cooling and lubricating drilling tools, transferring rock-breaking power [9–11], conveying hydraulic power, forming a filter cake to seal pores, assisting in the collection and interpretation of information, and enhancing the rate of penetration [12–14]. In the process of deep formation drilling, the performance of drilling fluid is often compromised by high temperature, high pressure and high salinity. These can lead to wellbore collapse, a stuck pipe, lost circulation and even blowout. Therefore, drilling fluid needs to have excellent temperature and salt resistance to guarantee drilling safety [15–17]. At present, water-based drilling fluids (WBFs) and oil-based drilling fluids (OBFs) are commonly used in drilling. Compared with WBFs, OBFs have better temperature resistance and lubricity. However, the base oil of OBFs will produce toxic aromatic hydrocarbons during use, so the environmental acceptance of OBFs is poor. In order to make the drilling fluids have both the high operational performance of OBFs and the environmental acceptance of WBFs, synthetic-based drilling fluids (SBFs) [18–21] came into being as an emerging family of drilling fluids. The continuous phase of SBFs is synthetic organic matter, which leads to its high cost and limits its popularization and application. Overall, WBFs have the lowest cost and the best environmental acceptance. Therefore, high temperature- and salt-resistant WBFs have always been an important consideration in drilling fluid research. They are mainly water, bentonite, viscosifier, filtrate reducer and weighting material. The filtrate reducer is the core additive of WBFs and is important for reducing the penetration of drilling fluid into formations and maintaining wellbore stability [22,23]. This requires higher standards for temperature and salt resistance of the filtrate reducer.

Globally, scholars have done much research on improving the temperature and salt resistance of filtrate reducers. The common filtrate reducers in oil and gas engineering are modified natural materials and synthetic polymers. Natural materials [24–26] have the attractions of wide availability, low cost and low environmental impact. Natural plant materials, such as starch, cellulose and lignin, have been modified by etherification and grafting to improve their water solubility and temperature resistance. Several mature industrial products have become established, such as polyanionic carboxymethyl cellulose (CMC), hydroxyethyl cellulose (HEC) and hydroxyethyl starch (HPS). However, the intrinsic characteristics of natural materials limit their applicability to temperatures below 150°C, which is insufficient for deep well drilling. With the deepening of research, researchers found that different groups in the polymer can give different properties to the polymer, such as the adsorption properties of amide groups, the hydration ability of carboxyl-enhanced molecules and the salt resistance of sulfonic acid groups. So, researchers hoped to achieve the desired effect by designing the structure of polymer. In molecular design, researchers select C–C and C–N bonds in the main chains and introduce functional monomers to improve the polymer filtrate reducer's temperature and salt resistance. Functional monomers endow the filtrate reducer with excellent properties. Many research results showed that compared with natural filtrate reducer materials, synthetic polymer filtrate reducer can be used at higher temperatures. For example, Ma *et al*. [27] used acrylamide (AM), 2-acrylamide-2-methylpropane sulfonic acid (AMPS) and N-vinylpyrrolidone (NVP) as monomers to synthesize a high temperature, salt-resistant and calcium-resistant polymer filtrate reducer, whose temperature resistance was 160°C and calcium resistance was 1.0 wt%. Using AMPS, N, N-diethylacrylamide (DEAM), dimethyl diallyl ammonium chloride (DMDAAC) and NVP, Huang *et al*. [28] synthesized a polymer filtrate reducer with a temperature resistance of 240°C. Although the polymer filtrate reducers have good temperature resistance and salt tolerance due to the polyelectrolyte effect of the polymer, the polymer molecular chains curl when exposed to NaCl, significantly reducing the drilling fluid's viscosity and increasing its filtration. More research is needed to improve polymer filtrate reducers' temperature and salt resistances further.

Nanomaterials have become a popular research topic in recent years because they have high activity, strong rigidity and good thermal stability. Studies showed that nanomaterials in the synthesis of filtrate reducers could improve their high temperature and salt resistances [29–32]. Wang *et al*. [33] prepared a nanocellulose filtrate reducer that reduced the filtration of 25.0 wt% brine-based mud to 18.6 ml after hot rolling at 180°C with the dosage of 2.0 wt%. Its performance was better than that of sulfonated lignite (SMC). Mao *et al*. [34] prepared a nanofiltrate reducer by compounding nanosilica with a hydrophobic associative polymer with excellent filtration reduction. When the dosage was 1.0 wt%, the high-temperature and high-pressure filtration of the freshwater-based mud was only 20.5 ml after ageing at 200°C. The mechanism analysis shows that the nanomaterials themselves have strong rigidity and thermal stability, so they can enhance the thermal stability of the filtrate reducer molecule. Under the conditions of high temperature and high salt, the molecular structure of the nanocomposite filtrate reducer remains intact, so that the polymer part can continue to play an important role. At the same time, in the process of forming the filter cake, nanoparticles can be stacked with each other to block the micro pores and micro fractures of formation rocks and reduce the filtration. Macroscopically, nanomaterials improve the temperature and salt resistance of the filtrate reducer.

Nano-laponite [35–37] is a synthetic layered trioctahedral silicate mineral with very fine particle size and nano characteristics. Its nanosheet layer of positively charged edges and negatively charged surface attract each other, which can form a ‘card house’ structure in aqueous solution. Nano-laponite has excellent thixotropy, thickening and adsorption properties. There are reports of nano-laponite being used as a drilling fluid mud material [38], viscosifier [39,40] and shale inhibitor [41,42]. However, there are few studies on the filtration performance of nano-laponite. Compounding nano-laponite with polymers to synthesize nanocomposites has good research prospects. To this end, the authors first modified nano-laponite [43,44], then used AMPS, AM, DMDAAC and γ-methacryloyloxypropyl trimethoxysilane (KH570) modified nano-laponite as reaction monomers to develop a nanocomposite filtrate reducer (ANDP) through free-radical aqueous solution copolymerization, and studied its high temperature and salt resistance in WBFs.

## 2. Experimental section

### 2.1. Materials

Acrylamide (AM, AR), 2-acrylamide-2-methylpropane sulfonic acid (AMPS, AR) and dimethyl diallyl ammonium chloride (DMDAAC, 60% aqueous solution) were purchased from Aladdin Reagent Co., Ltd. *γ*-Methacryloyloxypropyl trimethoxysilane (KH570, AR), ammonium persulfate (AR), absolute ethanol (AR) and sodium hydroxide (CP, 99.5%) were purchased from Sinopharm Chemical Readditive Co., Ltd. (Shanghai, China). Nano-laponite (20 nm inorganic nanoparticles) was purchased from Guangzhou Bofeng Chemical Technology Co., Ltd. The slurry bentonite used for WBFs was purchased from Huaian Tengfei Bentonite Development Co., Ltd., China.

### 2.2. Synthesis of nanocomposite filtrate reducer

Due to the protonation of Mg-OH in nano-laponite flakes, the edges of the nano-laponite particles become positively charged in water (pH < 11). Hydrolysis of KH570 in an aqueous ethanol solution produces Si-OH, which combines with the asymmetric stretching vibration peak of ^-^OH at the edges of nano-laponite to modify the nano-laponite [45–47].

Thirty millilitres of absolute ethanol and 10 ml deionized water were mixed thoroughly in a beaker, and the pH was adjusted to 4 with dilute HCl solution. Two grams of nano-laponite were added to the beaker and ultrasonically dispersed for 10 min. After this, 2 g KH570 was added to the beaker and completely mixed. The mixture in the beaker was transferred to a flask for continuous stirring and reacted in a water bath at 70°C for 8 h. The solid product was separated by centrifugation, washed with anhydrous ethanol two or three times, fully dried at 50°C, then ground in a mortar to form a white powder of modified nano-laponite.

**Scheme 1.**
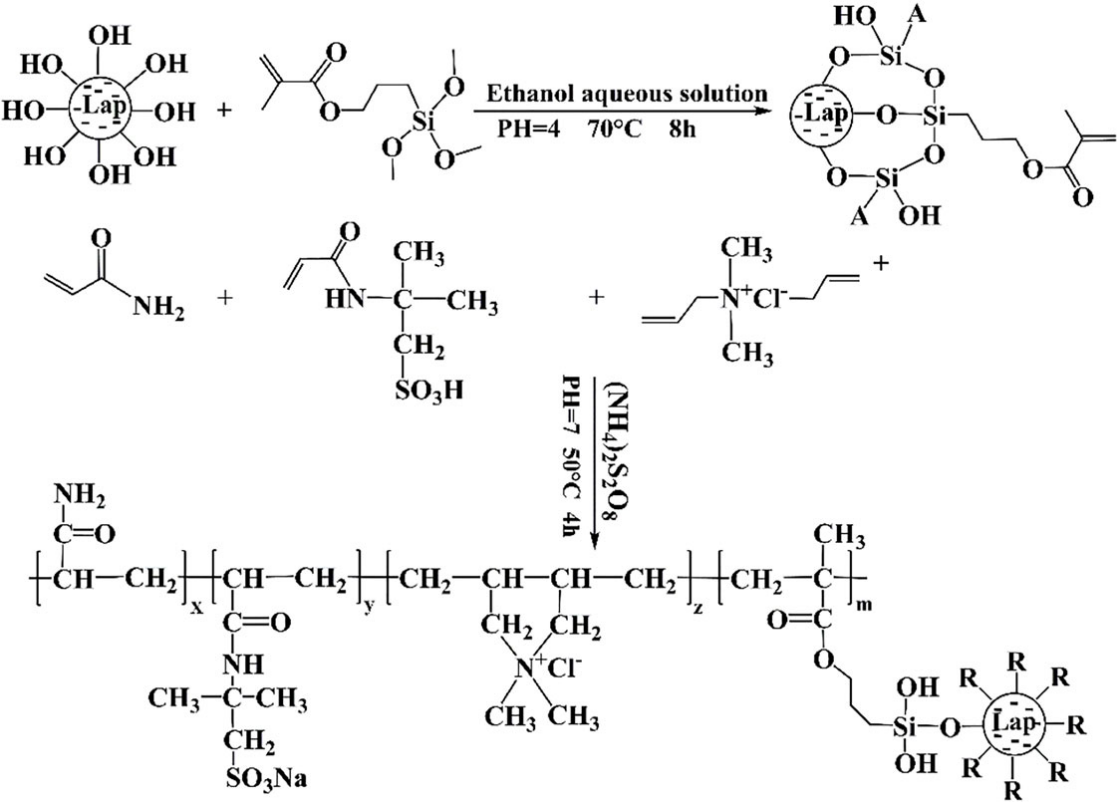
The synthesis procedure and molecular structure of ANDP.

In total, 20.66 g AMPS, 2.03 g AM and 2.31 g DMDAAC were mixed evenly in a beaker containing 75 g deionized water, with the pH adjusted to 7 with dilute NaOH solution. Next, 0.25 g modified nano-laponite was dissolved in 10 ml ethanol and ultrasonically oscillated for 10 min. The two solutions were then mixed thoroughly in a flask. After nitrogen was introduced into the flask, 0.05 g ammonium persulfate was added into the mixture and reacted in a water bath at 50°C for 4 h. It was purified with acetone and ethanol, dried at 50°C, and pulverized to obtain the final product: ANDP. The synthesis process and possible molecular structure of ANDP are shown in scheme 1.

### 2.3. Characterization of nanocomposite filtrate reducer

#### 2.3.1. Fourier transform infrared (FT-IR)

The synthetic product was characterized by the potassium bromide (KBr) tableting method using an infrared spectrometer. A small amount of synthetic product and KBr were mixed and ground into powder in a mortar, and the ground powder was pressed into a complete and transparent sheet by a tablet press. The scanning wavelength range was from 400 cm^−1^ to 4000 cm^−1^.

#### 2.3.2. Thermogravimetric analysis (TGA)

The thermal stability of ANDP was measured with a thermal analyzer (TGA550, Mettler Toledo Co.,) in an N_2_ environment. The experimental temperature range was 50.0°C to 1000.0°C, and the heating rate was 10.0°C min^−1^.

#### 2.3.3. Transmission electron microscopy (TEM) analysis

For TEM analysis, 1.0 wt% ANDP solution was prepared and ultrathin carbon film was selected as the supporting film. The 1.0 wt% ANDP solution was dropped onto an ultrathin carbon film and dried with an infrared lamp. The test voltage was 200 kV, and the morphology was observed using a JEM 2100F type transmission electron microscope.

### 2.4. Zeta potential and particle size analysis

The Zeta potential of drilling fluid samples was measured by a nanoparticle potentiometer (Zetasizer Nano Z, Malvern Instruments Co., Ltd.). The particle size of drilling fluids was measured on a particle size analyzer (Mastersizer 3000, Malvern Instruments Co., Ltd.).

### 2.5. Base mud preparation and drilling fluids performance testing

Sixteen grams of bentonite and 0.56 g sodium carbonate were dispersed uniformly in 400 ml water, stirred at a speed of 10 000 rpm for 20 min and then stood for hydration for 24 h to obtain the base mud.

The rheology and gel strengths (10 sec/10 min) of drilling fluids were tested by a six-speed rotary viscosimeter (ZNN-D6, Haitongda Co., Ltd., Shandong, China). The following equations calculate the apparent viscosity (AV), plastic viscosity (PV), dynamic shear (YP) and gel strengths (G′ and G′′):

2.1 $$\mathrm{AV}=\frac{\theta_{600}}{2}(\;\mathrm{mPa}\cdot s),$$

2.2 $$\mathrm{PV}=\theta_{600}-\theta_{300}\;(\;\mathrm{mPa}\cdot s),$$

2.3 $$\mathrm{YP}=(\;\theta_{300}-\mathrm{PV})\;/2\;(\;\mathrm{Pa}),$$

2.4 $$G^{\prime }=0.5\;\times \theta_{3}(\;10s,\mathrm{Pa})\;$$

2.5 $$\mathrm{and}\;G^{\prime \prime }=0.5\;\times \theta_{3}(10\;\min,\mathrm{Pa}).$$

where *θ*_600_, *θ*_300_ and *θ*_3_ are the values of the rotary viscometer at the corresponding speed. The gel strengths *G*′ and *G*″ are the shear force when the drilling fluids are stood for 10 s and 10 min, respectively.

According to American Petroleum Institute (API) standards, the API filtration of drilling fluids was measured by medium-pressure filtration meter (SD, Qingdao Tongchun Petroleum Instrument Co., Ltd.). The API filtration of drilling fluids is the volume of filtrate measured within 30 min under a fixed pressure of 100 psi. The API filtration and rheology of drilling fluids were measured before and after ageing. The high-temperature and high-pressure (HTHP) filtration was measured on a high-temperature (the hot rolling temperature) and high-pressure (3.5 ± 0.03 MPa) filtration apparatus (GGS42-2, Qingdao Tongchun Petroleum Instrument Co., Ltd.).

### 2.6. Permeability and micromorphology of drilling fluid filter cake

After the API filtration measurement, the wet mud cake was removed from the kettle body of the medium-pressure filtration meter carefully and completely, and the thickness was measured. The permeability (*K*) of a fresh mud cake [48] was calculated according to Darcy's formula

2.6 $$K=\frac{\mu tq}{\Delta PA},$$

where *μ* is the viscosity of the filtrate (1 cP); Δ*P* is the pressure drop (6.8 atm); *t* is the thickness of the mud cake (cm); *A* is the area of the filter paper (45.8 cm^2^); and *q* is the filtrate rate (cm^3^ s^−1^), which can be calculated by the ratio of filtration to measurement time. Specifically, by adding 100 ml deionized water into the sample cup of the medium-pressure filter and allowing the deionized water to flow out through the mud cake under 100 psi pressure. The filtrate volume was recorded every 180 s and plotted with time to fit a straight line. The slope of the straight line corresponded to the *q* value.

The microscopic morphology of the dried mud cake was observed with a scanning electron microscope (SEM, EVO-LS15, ZEISS, DE).

## 3. Results and discussion

### 3.1. Characterization of nanocomposite filtrate reducer

#### 3.1.1. FT-IR spectroscopic analysis

**Figure 1.**
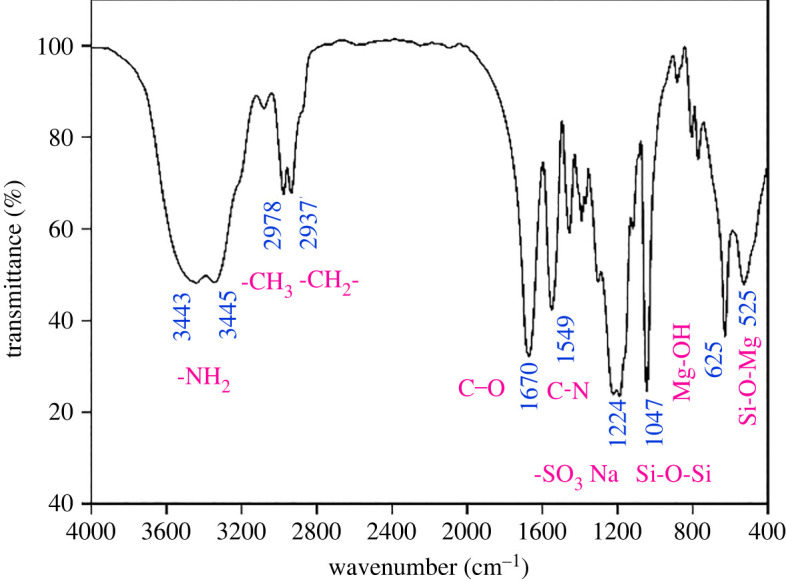
Infrared spectrum of ANDP.

As shown in figure 1, the doublet peaks at 3443 cm^−1^ and 3345 cm^−1^ are the antisymmetric and symmetric tensile vibration absorption peaks of two N–H bonds of the ^-^NH_2_ group in AM, respectively. The wavelength of 2978 cm^−1^ is the asymmetric stretching vibration peak of ^-^CH_3_. The peak at 2937 cm^−1^ is the stretching vibration peak of the C–H bond in methylene. The peak at 1670 cm^−1^ is a strong stretching absorption peak of the C=O bond in the amide group. The characteristic absorption peak of the C–N bond in DMDDAC is the absorption peak at 1549 cm^−1^. The characteristic wavelength of 1224 cm^−1^ is the absorption peak of the -SO_3_Na group in AMPS. The absorption peak at 1047 cm^−1^ is the asymmetric absorption peak of Si-O-Si in KH570-modified nano-laponite. The peaks at 625 cm^−1^ and 525 cm^−1^ are the characteristic absorption peaks of nano-laponite [38,45]. The peak value of 625 cm^−1^ is the bending vibration peak of Mg-OH, and 525 cm^−1^ is the deformation vibration peak of Si-O-Mg. The infrared spectra show that the molecular chains of ANDP contain all reactive monomers.

#### 3.1.2. Thermogravimetric analysis

**Figure 2.**
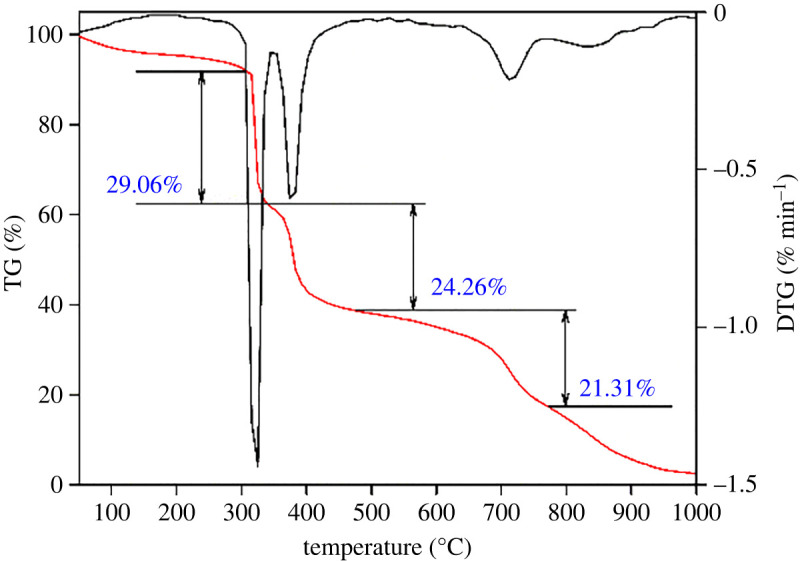
Thermogravimetric analysis curve.

Figure 2 shows the three pronounced peaks in the derivative thermogravimetry (DTG) curve corresponding to the three thermal weightlessness platforms of the thermogravimetry (TG) curve. The weight loss process can be divided into five stages. In the first stage, from 50°C to 306°C, the TG curve decreases slowly, and the weight loss rate is 7.84%. The mass loss in this stage is due to the thermal volatilization of water in the sample. On the one hand, the sample has strong hydrophilicity, making it easy to absorb free moisture. On the other hand, the molecular structure contains strong hydrophilic groups, such as the amide group in AM and sulfonic acid group in AMPS, that easily adsorb free water molecules in the environment and form intramolecular-bound water through interaction. In the second stage, from 306°C to 335°C, the weight loss rate is 29.06%. In this stage, due to the thermal decomposition of amide groups [49], the TG curve decreases sharply, and the thermal decomposition rate reaches the highest at 324°C. In the third stage, when the temperature ranges from 335°C to 470°C, there is a 24.26% thermal weightlessness platform, and the mass loss is noticeable. The decomposition rate reaches a maximum at 382°C, corresponding to the second peak of the DTG curve.

During this stage, the sulfonic acid group begins to decompose rapidly. The main chain and a side chain of the molecular structure also begin to decompose and break at the same time [28]. In the fourth stage, the temperature ranges from 470°C to 770°C. The thermal weightlessness is 21.31%. During this stage, the main chain and the side chain of the molecular structure continue to decompose and break. The DTG curve shows the third peak at 712°C due to the thermal decomposition of crystal water and the phase transformation of nano-laponite [39]. In the fifth stage, when the temperature ranges from 770°C to 1000°C, the remaining material of the polymer sample is carbonized and decomposed continuously. Analysis of the thermogravimetric experimental result shows that the thermal decomposition of ANDP begins at 306°C, indicating that ANDP has good thermal stability. Generally, the geothermal gradient in most parts of the world is 30°C km^−1^, and the depth of large oil and gas reservoirs distributed in deep and ultra-deep strata can reach 6000 m. At this depth, the bottom-hole temperatures are close to 200°C. The thermal decomposition temperature of ANDP is higher than the bottom-hole temperature, indicating that ANDP can be used in HTHP drilling environments.

#### 3.1.3. TEM analysis

**Figure 3.**
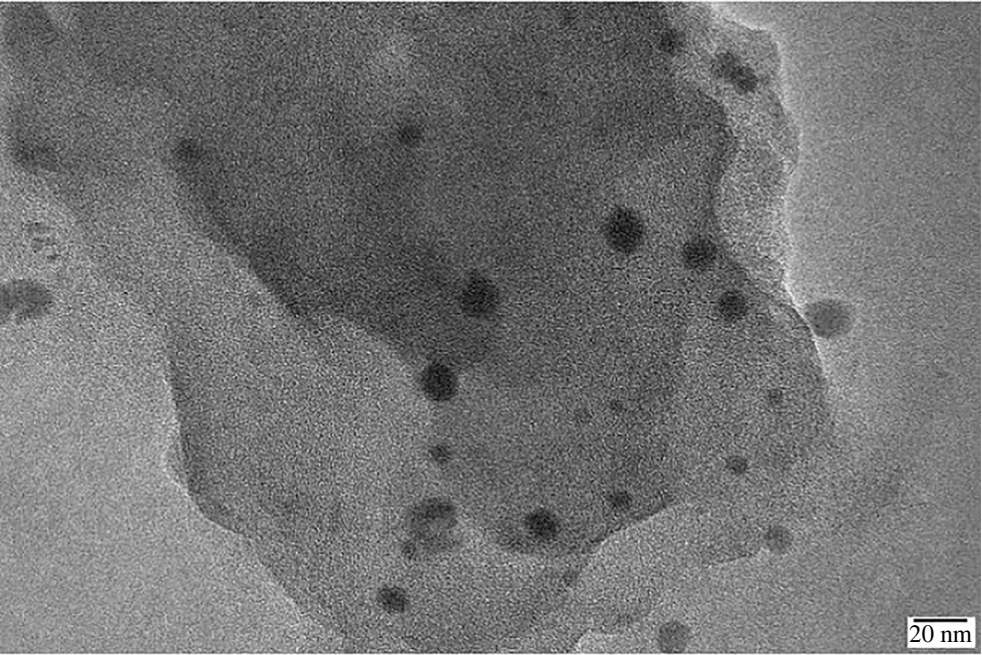
Transmission electron microscope image.

Nanomaterials, such as nano silicon dioxide and nano calcium carbonate, are prone to agglomeration in solvents due to their large specific surface area and high reactivity. When used as reaction monomers, they tend to be unevenly dispersed in the reaction solvent, thus affecting the reaction process [50]. However, nano-laponite has excellent dispersion characteristics in aqueous solution, and will not agglomerate and affect the progress of polymerization. Figure 3 shows that there is modified nano-laponite in the molecular structure of ANDP, and the average diameter of modified nano-laponite particles is 20 nm. This indicates that the modified nano-laponite sample had good dispersibility and successfully participated in the reaction.

### 3.2. Performance of nanocomposite filtrate reducer

#### 3.2.1. Temperature resistance

**Figure 4.**
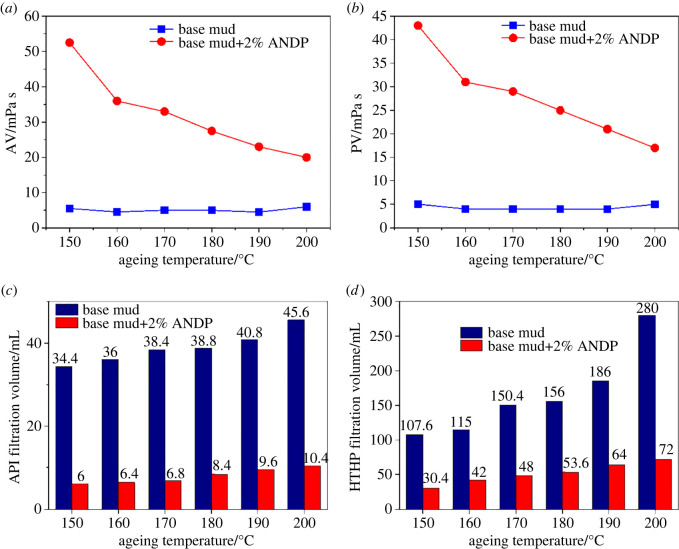
Effect of temperature on the (*a*) AV, (*b*) PV, (*c*) API filtration and (*d*) HTHP filtration values of drilling fluids.

To investigate the temperature resistance of ANDP, 2.0 wt% ANDP was added to freshwater-based mud to prepare drilling fluids, and hot rolling experiments were carried out at different temperatures. After hot rolling for 16 h, the rheological parameters, API filtration and gel strengths of the drilling fluids were measured after cooling. The HTHP filtration was measured at the corresponding hot rolling temperature. The rheological parameters, filtration results and gel strengths are shown in figure 4 and table 1.

**Table 1.** Effect of temperature on the YP, dynamic plastic ratio and gel strengths of drilling fluids.

ageing temperature/°C	*YP*/Pa		YP/PV		*G*′/Pa		*G*″/Pa	
	base mud	ANDP	base mud	ANDP	base mud	ANDP	base mud	ANDP
150	0.5	9.5	0.10	0.22	0	0.5	0.5	1.0
160	0.5	5.0	0.13	0.16	0	0.5	0	0.5
170	1.0	4.0	0.25	0.14	0	0.5	0	0.5
180	1.0	2.5	0.25	0.10	0	0	0	0.5
190	0.5	2.0	0.13	0.10	0	0	0	0
200	1.0	3.0	0.20	0.18	0	0	0	0

As the ageing temperature increased, the AV (figure 4*a*), PV (figure 4*b*) and YP of the base fluid were very low and remained almost unchanged. After ageing, the gel strengths of the base fluid were 0 Pa, indicating that the gel reticular structure inside the base fluid had been completely destroyed. The API filtration (figure 4*c*) and HTHP filtration (figure 4*d*) increased significantly. The HTHP filtration at 200°C was as high as 280 ml. Compared with the base mud, the gel strengths of the drilling fluids with 2.0 wt% ANDP added were higher than that of the base mud, indicating that the addition of ANDP enhanced the gel reticular structure in the drilling fluids. The dynamic plastic ratios (YP/PV) of base mud and the drilling fluids with 2.0 wt% ANDP were small, indicating that the thixotropy was weak. At high temperature, the drilling fluids with 2.0 wt% ANDP maintained a higher viscosity at high temperatures and significantly reduced the filtration. After ageing at 200°C, the AV was 20 mPa s, the API filtration was only 10.4 ml and the HTHP filtration was 72 ml. The comparison indicates that ANDP still had good filtration performance at 200°C. Because the DMDAAC in ANDP molecules can form a rigid ring structure, the nano-laponite has strong temperature resistance. The main chain is a C−C bond with high bond energy, which is not easy to break so that ANDP is not easily degraded at high temperatures. In addition, nano-laponite can be electrically adsorbed with clay particles, and the amide group in the molecule can also be adsorbed on the surface of clay particles through hydrogen bonding. Therefore, ANDP molecules can be firmly adsorbed with clay particles under the dual action of hydrogen bonding and electrostatic adsorption [2]. ANDP does not degrade easily and it is not easy to desorb from clay particles, which are the guarantees for the filtrate reducer to play a role under high-temperature conditions.

#### 3.2.2. Salt resistance

**Figure 5.**
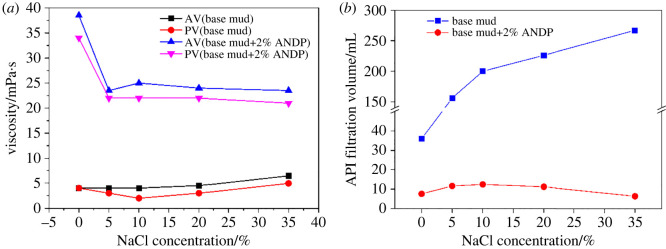
Effect of NaCl concentration on (*a*) viscosity and (*b*) API filtration of drilling fluids.

For the study of salt resistance, 2.0 wt% ANDP was added to freshwater-based mud. After stirring for 20 min at 4000 rpm and standing at room temperature for 24 h, different concentrations of NaCl were added to prepare drilling fluids. After ageing at 150°C for 16 h, the rheological parameters, API filtration and gel strengths of the drilling fluids were measured after cooling. The rheological parameters, filtration results and gel strengths are shown in figure 5 and table 2. The experimental results revealed the salt resistance of ANDP.

**Table 2.** Effect of NaCl concentration on the YP, dynamic plastic ratio and gel strengths of drilling fluids.

NaCl concentration/%	*YP*/Pa		YP/PV		G′/Pa		G″/Pa	
	base mud	ANDP	base mud	ANDP	base mud	ANDP	base mud	ANDP
0	0	4.5	0	0.13	0	0.5	0.5	1.0
5	1.0	1.5	0.33	0.07	0	0	0	0.5
10	2.0	3.0	1.00	0.14	0	0	0.5	1.0
20	1.5	2.0	0.50	0.09	0	0	0	0.5
35	1.5	2.5	0.30	0.12	0	0	0	0.5

**Figure 6.**
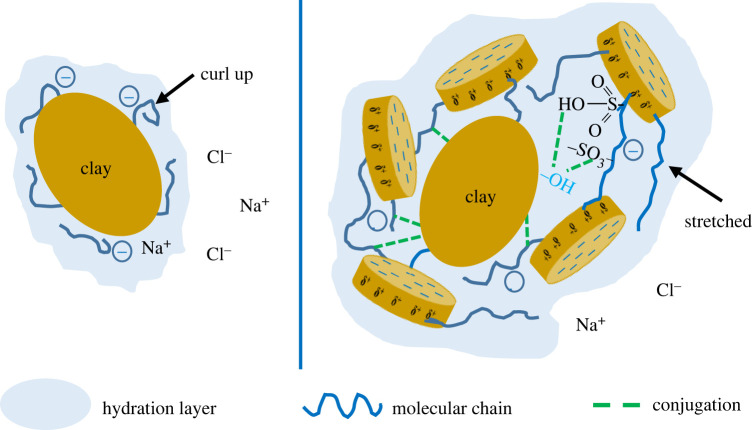
ANDP improves the performance of salt-bearing drilling fluids.

Figure 5 and table 2 show that after ageing at 150°C, with increased salt concentration, the viscosity (figure 5*a*) and YP of the base fluids remained low, the gel strengths were as low as 0 Pa, and the filtration (figure 5*b*) increased sharply. For a salt concentration of 35 wt%, the filtration of base mud was as high as 267 ml. Compared with the base mud, even with the increased salt concentration, the drilling fluids with 2.0 wt% ANDP added still had a certain gel strength, indicating that the addition of ANDP maintained the gel reticular structure of the drilling fluids under high-temperature and high-salt conditions. The drilling fluids with 2.0 wt% ANDP still maintained high viscosity and low filtration. When the salt concentration was 35 wt%, the AV of the drilling fluids was 23.5 mPa s, and the API filtration was only 6.4 ml, indicating that ANDP still had a good filtration reduction effect at 150°C and under saturated salt conditions. The dynamic plastic ratios of base mud varied widely, while the dynamic plastic ratios of drilling fluids with 2.0 wt% ANDP basically maintained a low value, indicating that the thixotropy of all was poor under high-temperature and high-salt conditions. The thixotropy of drilling fluids with 2.0 wt% ANDP maintained a weak stable state under the action of ANDP. The ring structure of DMDAAC and the nano-laponite layer contained in the molecule improved the rigidity of the molecular chain. The rigid molecular chains of ANDP did not easily curl up in the salt-bearing drilling fluids and maintained a stretching state, thus improving the viscosity of the drilling fluids. In addition, the molecular chains of ANDP contained many sulfonic acid groups with excellent temperature and salt resistance, which were insensitive to salts. The sulfonic acid groups formed a stable conjugated system [6,28] with hydrophilic groups such as hydroxyl groups to prevent the intrusion of salt ions, which ensured the effect of ANDP under high-salt conditions and improved the overall salt resistance of the drilling fluids. In combination with figure 6, the mechanism of ANDP improving the performance of drilling fluid under saline conditions can be better understood.

### 3.3. Mechanistic investigation

#### 3.3.1. Zeta potential analysis

The Zeta potential of base mud and drilling fluids containing 2.0 wt% ANDP at different NaCl concentrations were measured after hot rolling at 150°C.

**Figure 7.**
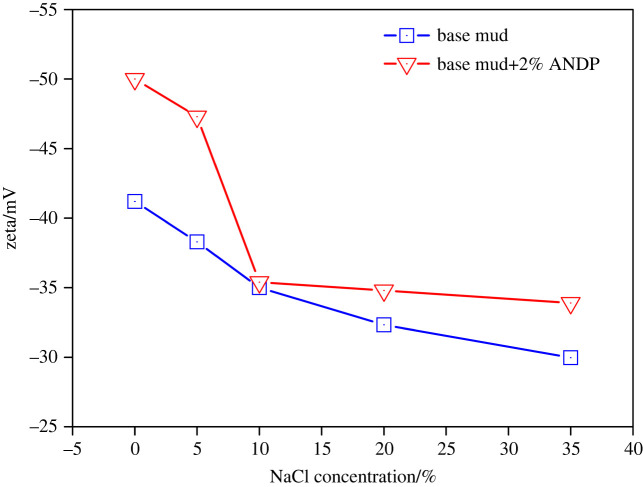
Effect of NaCl concentration on the Zeta potential of drilling fluids.

Figure 7 shows the influence of NaCl concentration on the Zeta potential of base mud and drilling fluids containing 2.0 wt% ANDP. As the concentration of NaCl increased, the absolute value of the Zeta potential of base mud and drilling fluids with 2.0 wt% ANDP gradually decreased. Under different NaCl concentrations, the absolute values of the Zeta potential of drilling fluids with 2.0 wt% ANDP were higher than that of base mud. The results showed that the addition of NaCl reduced the stability of the clay dispersion system. With the increase of NaCl concentration, the diffusion electric double layer of bentonite particles was continuously compressed, the hydration layer of clay particles became thin and the absolute value of the Zeta potential gradually decreased. In the drilling fluids containing ANDP, ANDP molecules were firmly adsorbed with clay particles, making the hydration layer thicker. The thickening of the hydration film of clay particles led to an increase in both the negative charge on the clay particles and the absolute value of the Zeta potential. The macromolecule chains of ANDP shielded and reduced the influence of NaCl on clay particles, which is helpful to enhance the colloidal stability of the drilling fluid dispersion system. The Zeta potential is an important indicator to characterize the stability of the colloidal dispersion systems. Usually, the absolute value of the Zeta potential is greater than 30 mV, indicating that the dispersion system is stable [1,10]. The absolute values of the Zeta potential of drilling fluids containing ANDP were greater than 35 mV at different salt concentrations, indicating that the colloid stability of the drilling fluid is good and that ANDP protected the colloidal stability of the drilling fluid dispersion system under high-temperature and high-salt conditions.

#### 3.3.2. Particle size analysis

**Figure 8.**
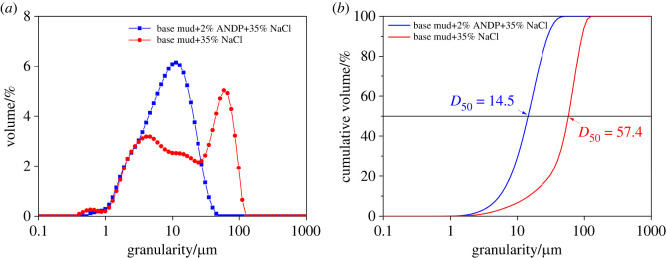
(*a*) Granularity distribution diagram and (*b*) cumulative volume distribution diagram of drilling fluids.

For particle size analysis, 35 wt% NaCl was added to the freshwater base fluid and the drilling fluid containing 2.0 wt% ANDP. After ageing at 150°C for 16 h, the particle size distributions of base mud and drilling fluid containing 2.0 wt% ANDP were measured, and the results are shown in figure 8.

Figure 8 shows that the median particle size D_50_ of saturated brine-based mud (35 wt% NaCl) after hot rolling was 57.4 µm. The particle size was large and the size distribution was noticeably polarized. The median particle size D_50_ of saturated brine drilling fluid containing 2.0 wt% ANDP was 14.5 µm and the particle size distribution was relatively concentrated. The peak particle size was 16 µm and the overall particle size was smaller than that of saturated brine-based mud.

The reasons for the above experimental results were analysed. First, under the influence of high temperature and high salt, the hydration layer on the surface of clay particles was compressed. When the hydration layer of clay particles was compressed and became thin, the repulsion between clay particles decreased. Therefore, clay particles tended to agglomerate, resulting in larger particles. The polarized distribution of hydrated clay particle size led to the poor quality of filter cake, which increased the filtration. ANDP molecules were adsorbed with clay particles, making the hydration layer of clay particles thicker and weakening the aggregation of clay particles [3,27]. Second, there were multiple adsorption sites on the ANDP molecular chains and multiple clay particles were adsorbed at the same time under the action of bridging, forming a complex network structure full of the whole drilling fluid system [51]. The complex network structure improved the aggregation stability of clay particles and was beneficial to maintaining the content of fine particles in drilling fluids. Finally, under the protection of ANDP, the clay particles were smaller and the particle size distribution was more reasonable. The size of clay particles was normally distributed without polarization and the formed filter cake was more compact, which significantly reduced the filtration of drilling fluids.

#### 3.3.3. Permeability and microscopic appearance of filter cake

**Figure 9.**
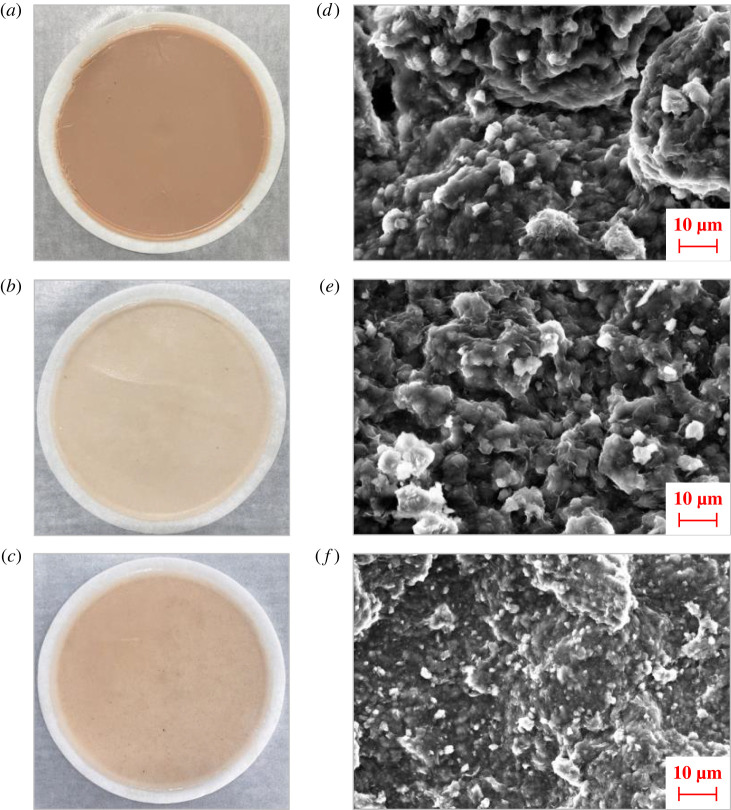
Filter cake and SEM images (3000×) of base mud (*a*,*d*), base mud with 1.0 wt% ternary filtrate reducer (*b*,*e*), and base mud with 1.0 wt% ANDP (*c*,*f*).

On the basis of synthesizing ANDP, the nano-laponite modified by KH570 was removed and the ternary filtration reducer was synthesized. In a flask containing 75 g deionized water, 20.66 g AMPS, 2.03 g AM and 2.31 g DMDAAC were uniformly mixed and the pH was adjusted to 7 with dilute NaOH solution. Nitrogen gas was introduced into the flask, 0.05 g ammonium persulfate was added and the reaction was carried out in a water bath at 50°C for 4 h. After the reaction, the product was purified with acetone and ethanol, dried at 50°C, and crushed to obtain the ternary filtration reducer. ANDP has better filtration reduction performance than ternary filtration reducer. In order to further analyze the mechanism of ANDP, 1.0 wt% ANDP was added to a cup of freshwater-based mud and 1.0 wt% ternary filtrate reducer was added to another cup of freshwater-based mud to prepare two kinds of drilling fluids. After ageing at 150°C, the mud cake was obtained after measuring the filtration volume according to the API standard and its permeability was measured. The microstructure of the dried filter cake was observed by SEM. The SEM imaging and filter cake parameters of the base mud, the base mud with 1.0 wt% ternary filtrate reducer added, and the base mud with 1.0 wt% ANDP added are shown in figure 9 and table 3.

**Table 3.** Determination of different filter cake parameters.

filter cake sample	base mud	base mud with ternary filtrate reducer	base mud with ANDP
*t*/cm	0.40	0.08	0.07
*q*/(10^–3^ cm^3^ s^−1^)	9.23	3.33	2.78
*K*/(10^–3^ mD)	13.69	0.99	0.72

In the drilling process, the quality of the filter cake will also affect the filtration performance of the drilling fluid, so this is also an important evaluation index of the performance of the drilling fluid system [52,53]. The base mud filter cake (figure 9*a*) was the thickest. The filter cake of base mud with ternary filtrate reducer (figure 9*b*) was thin and the surface was rough. The filter cake of base mud with ANDP (figure 9*c*) was relatively thin and tough with a smooth surface. From the SEM image of the base mud filter cake (figure 9*d*), it can be seen that the clay particles agglomerate and pile up to form large clay particles, and there are many microcracks and microvoids on the surface of the filter cake. After high-temperature ageing, due to the effect of high-temperature dehydration, the hydration degree of clay particles decreased and the hydration layer became thinner. The electrostatic repulsion between the clay particles was reduced, leading to agglomeration between them. The clay particles were piled together, which in turn formed larger clay particles. Many microcracks and microvoids led to increased permeability of the filter cake, which was the main reason for the decrease of drilling fluid system performance and the increase of filtration after high-temperature ageing.

The surface of the filter cake of the base mud with ternary filtrate reducer (figure 9*e*) was uneven and there were large clay particles. Some of the particles were agglomerated. There were no large fissures, but there were more microcracks and fine voids. There were no obvious cracks or voids on the surface of the filter cake of the base fluid with ANDP (figure 9*f*). Many fine clay particles were tightly packed to form a dense mud cake, which significantly improved the quality of the filter cake. This demonstrated that under the action of ANDP, the clay particles maintain better hydration after high-temperature ageing and good dispersion, and inhibit the high-temperature aggregation of clay particles. This further reflects that ANDP had good high-temperature resistance and filtration-reduction effect.

The flow parameter *q* is the slope of the straight line of the filtration volume versus time and, according to Darcy's law, the permeability of the filter cake was calculated as shown in table 3.

As shown in table 3, the permeability of the base mud filter cake was the highest and the permeability of the filter cake decreased significantly after adding a ternary filtrate reducer. The permeability of filter cake formed by adding ANDP to the base mud was the lowest. The measurement results of filter cake permeability were mutually verified with filter cake SEM images. The combination of the two experimental results shows that under the action of ANDP, the clay particles maintained an excellent dispersion and formed a denser mud cake, which significantly reduced the filtration.

#### 3.3.4. Mechanism of nanocomposite filtrate reducer

**Figure 10.**
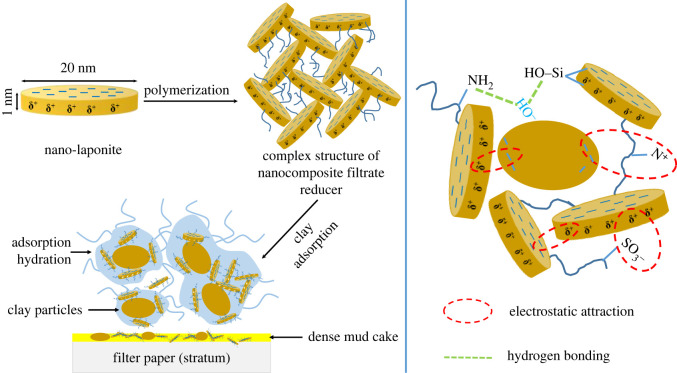
Mechanism of nanocomposite filtrate reducer (ANDP).

Integrating and analysing the above experimental results, the principles of nanocomposite filtrate reducer ANDP were obtained. In order to better describe the action mechanism of ANDP, it is schematically illustrated in figure 10. The action mechanism of ANDP can be summarized as follows:

1. In the molecular structure of ANDP, multiple molecular chains are connected to the modified nano-laponite, which acts as the physical cross-linking agent. The cross-linking structure improves the temperature and salt resistance of ANDP so that the drilling fluid can maintain its viscosity under high-temperature and high-salt conditions.
2. ANDP has good colloidal protection and can adsorb with clay particles so that the hydration layer on the surface of clay particles becomes thicker. Even under the conditions of high temperature and high salt, ANDP is not easy to desorb, which can effectively maintain the hydration degree of clay particles. In addition, the complex network structure of ANDP and the bridging effect with clay particles jointly improve the colloidal structure of the drilling fluid system. The colloidal protection of ANDP and the colloidal structure of the drilling fluid jointly improve the aggregation stability of clay particles, which is beneficial to maintain the content of fine particles in the drilling fluid and form a dense filter cake, thus reducing the filtration.

## 4. Conclusion

Comprehensively summarizing our research contents, the following three conclusions are drawn:

1. A nanocomposite filtrate reducer (i.e. ANDP) was successfully synthesized by a free-radical polymerization reaction in an aqueous solution using AMPS, AM, DMDAAC and KH570 modified nano-laponite. The TGA result shows that the initial decomposition temperature of an ANDP molecule is 306°C, which represents good thermal stability.
2. ANDP effectively maintains the viscosity of drilling fluid, reduces filtration, and has good temperature and salt resistance. When the ANDP is 2.0 wt%, the API filtration of the freshwater drilling fluid is 10.4 ml after hot rolling at 200°C for 16 h, and the API filtration of saturated brine drilling fluid is 6.4 ml after hot rolling at 150°C for 16 h. The feasibility of nano-laponite as a synthetic material for filtration reducer was verified.
3. The complex cross-linking structure of ANDP improves its temperature and salt resistance. ANDP also has a good adsorption capacity. After adsorption with clay particles, it has excellent colloidal protection, increases the complexity of the drilling fluid network structure, improves the aggregation stability of clay particles, maintains a reasonable particle size distribution of solid particles in drilling fluids, forms a more compact filter cake and significantly reduces filtration.

## Data Availability

Our data are deposited at Dryad: https://doi.org/10.5061/dryad.8kprr4xqn [54].
